# Supplementary material for: Identification and Toxicity Prediction of Biotransformation Molecules of Organophosphate Flame Retardants by Microbial Reactions in a Wastewater Treatment Plant
Source: Int J Mol Sci. 2021 May 20;22(10):5376. doi: 10.3390/ijms22105376 (PMC8160787; doi:10.3390/ijms22105376)
Supplement: Supplementary file 1 [file ijms-22-05376-s001.zip › ijms-1209702-supplementary.pdf]

Supplementary Materials

Identification and toxicity prediction of biotransformation molecules of organophosphate flame retardants by microbial reactions in wastewater treatment plants

Table S1. Time gradient of mobile phase B in ultra-high-performance liquid chromatography. Solvent A in the mobile phase was 0.1% formic acid with water, and solvent B was 0.1% formic acid with methanol.

Time	Flow (ml/min)	%B	Curve
0	0.2	10	5
4	0.2	50	5
17	0.2	95	5
25	0.2	95	5
25.1	0.2	10	5
29	0.2	10	5

Table S2. Predicted LC50 (50% of lethal concentration) in each aquatic species (algae, daphnid, fish). The $\frac{\mathbf{10^6}}{\sum \boldsymbol{LC50}}$ value was the calculated magnitude toxicity to represent Figure 5.

Unit (mg/L)

	Algae	Daphnid	Fish	10^6/∑LC50
TBEP	2.8.E+01	2.6.E+01	4.2.E+01	1.0.E+04
TB_88	1.4.E+03	2.6.E+03	5.0.E+03	1.1.E+02
TB_198	7.7.E+02	1.8.E+03	3.7.E+03	1.6.E+02
TB _298	1.6.E+02	2.3.E+02	4.2.E+02	1.2.E+03
TB _412	3.5.E+02	5.8.E+02	1.1.E+03	5.0.E+02
TB _414	3.4.E+02	5.7.E+02	1.0.E+03	5.1.E+02
TCPP	2.8.E+01	2.7.E+01	4.3.E+01	1.0.E+04
TC_90	2.1.E+04	7.9.E+04	1.8.E+05	3.6.E+00
TC_249	1.5.E+02	2.3.E+02	4.1.E+02	1.3.E+03
TC_290	3.1.E+03	9.4.E+03	2.0.E+04	3.1.E+01
TC_304	4.3.E+03	1.4.E+04	3.1.E+04	2.0.E+01
TPHP	1.5.E+00	7.0.E-01	1.0.E+00	3.1.E+05
TP_110	3.3.E-01	2.1.E-01	1.6.E-01	1.4.E+06
TP_138	1.3.E+02	2.1.E+02	4.0.E+02	1.4.E+03
TP_140	1.0.E+03	2.8.E+03	6.0.E+03	1.0.E+02
TP_188	9.1.E+03	2.3.E+04	4.7.E+04	1.3.E+01
TP_250	2.2.E+01	2.1.E+01	3.4.E+01	1.3.E+04
TP_342	3.5.E+00	2.0.E+00	2.9.E+00	1.2.E+05

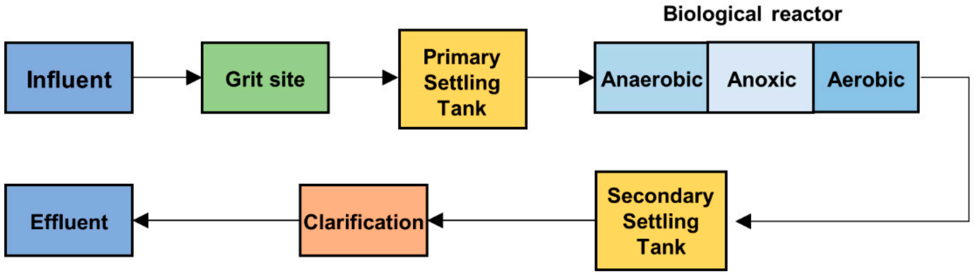

Figure S1. Overview of the wastewater treatment plant. Samples were collected in influent, after a biological reactor, and effluent.
